# Measuring biological age using a functionally interpretable multi‐tissue RNA clock

**DOI:** 10.1111/acel.13799

**Published:** 2023-03-16

**Authors:** Sascha Jung, Javier Arcos Hodar, Antonio del Sol

**Affiliations:** ^1^ Computational Biology Group CIC bioGUNE‐BRTA (Basque Research and Technology Alliance), Bizkaia Technology Park Derio Spain; ^2^ Department of Biochemistry and Molecular Biology University of the Basque Country (UPV/EHU) Leioa Spain; ^3^ Computational Biology Group, Luxembourg Centre for Systems Biomedicine (LCSB) University of Luxembourg Esch‐sur‐Alzette Luxembourg; ^4^ Ikerbasque, Basque Foundation for Science Bilbao Spain

**Keywords:** aging, machine learning, transcriptomics

## Abstract

The quantification of the biological age of cells yields great promises for accelerating the discovery of novel rejuvenation strategies. Here, we present MultiTIMER, the first multi‐tissue aging clock that measures the biological, rather than chronological, age of cells from their transcriptional profiles by evaluating key cellular processes. We applied MultiTIMER to more than 70,000 transcriptional profiles and demonstrate that it accurately responds to cellular stressors and known interventions while informing about dysregulated cellular functions.

## Graphical abstract

Quantifying the biological rather than the chronological age of cells has the potential to accelerate the identification of novel rejuvenation strategies. Here, we introduce MultiTIMER, a multi‐tissue RNA clock that measures the age of cells from their transcriptome thereby quantifying the activity of processes underlying the aging phenotype.

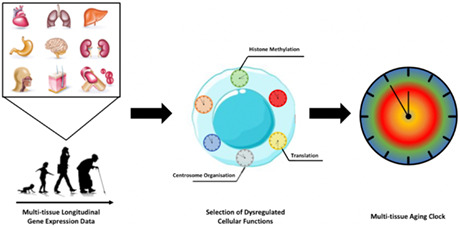

**Abbreviations**
BRAF: B‐Raf Proto‐Oncogene, Serine/Threonine Kinase
GLM: Generalized Linear Model
hTERT: human Telomerase Reverse Transcriptase
MAE: Mean Absolute Error
MBotC: Molecular Biology of the Cell
NIA: National Institute on Aging
RAS: Ras Proto‐Oncogene, GTPase
SV: Simian Virus
TMM: Trimmed Means of M Values

## 1 INTRODUCTION

Aging is a multifactorial process that is characterized by the progressive decline of cellular, tissue, and bodily functions. Although human lifespan has already significantly increased in the past century, great efforts are being devoted to characterize age‐related dysregulations in cellular processes, which could lead to disease onset, as well as to identify health‐promoting and rejuvenating interventions that can be preemptively administered (Ferrucci et al., 2020; Zhang et al., 2022). However, to characterize these age‐related dysregulations and determine suitable therapeutics, it is imperative to be able to quantify the ability of cells to perform their intended functions, a measure coined as biological age (Jazwinski & Kim, 2019). In contrast to chronological age, that is, the time a person has been alive, biological age is impacted by intrinsic and external factors, such as genomic aberrations, diet or stress, and molecularly reflected in nine hallmarks of aging (López‐Otín et al., 2013).

Due to the complexity of experimentally determining the integrity of cellular functions, several studies aimed at measuring the biological age of cells by developing computational aging clocks that use various molecular modalities, such as DNA methylation, proteomics or transcriptomics (Rutledge et al., 2022). Although these clocks have been shown to capture certain aspects of biological aging, they predominantly represent chronological age (Bell et al., 2019; Rutledge et al., 2022). Moreover, the interpretability of the molecular features found to be predictive of age remains limited. Although clocks based on transcriptional and proteomic data alleviated this issue, the selected genes and proteins are scattered across processes or are biomarkers, thus do not provide a comprehensive assessment of potentially impaired cellular functions.

## 2 RESULTS AND DISCUSSION

### 2.1 Development of a functionally interpretable RNA clock

To address these issues, we developed a functionally interpretable multi‐tissue RNA clock called MultiTIMER, that captures trends of biological aging of cells while providing insights into the individual processes declining throughout aging. MultiTIMER combines prior knowledge about gene‐function associations from the Molecular Biology of the Cell Ontology (MBotC) (Hansen et al., 2017) with a machine learning approach to identify a set of cellular processes and their associated genes whose expression is predictive of age. Contrary to existing approaches, this framework more directly connects aging and cellular functional decline in an unbiased manner, therefore allowing to measure biological age. In particular, we exploit the hierarchical organization of the MBotC and select the 27 top‐level processes that subsume more than 750 subprocesses and can potentially be linked to aging phenotypes, MultiTIMER identifies a subset of these processes that are most predictive of cellular age and thereby provides an unbiased assessment of the underlying cellular functions declining with age. In contrast, a previous approach named RNAAgeCalc (Ren & Kuan, 2020) aimed at predicting the age of cells from a set of 1600 genes that are consistently differentially expressed during aging across tissues. However, only a subset of these genes is enriched in cellular processes, which hinders the interpretability of how changes in the predicted age relate to cellular function. Another method aimed at addressing this shortcoming by predicting cellular age from a set of 50 hallmark gene sets representing specific well‐defined biological processes or states (Holzscheck et al., 2021). However, these gene sets represent only a small fraction of cellular processes affected during aging with several of them relating to tissue or cell type specific processes.

**FIGURE 1:**
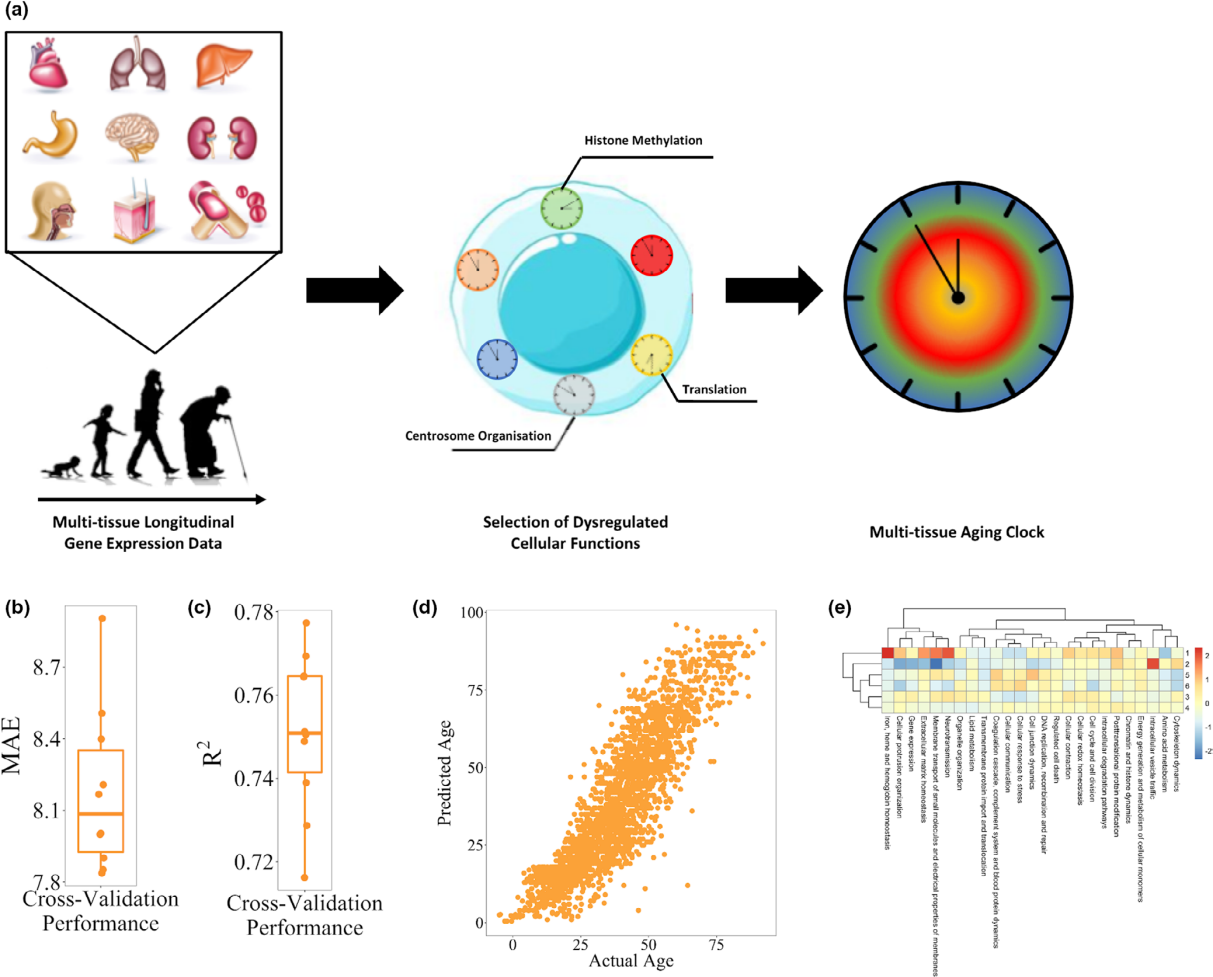
Design and validation of MultiTIMER. (a) Schematic overview of the MultiTIMER workflow. Starting from a multi‐tissue longitudinal transcriptomic dataset, cellular processes are identified that are most predictive of chronological age. All genes of these selected processes serve as an input for a generalized linear model that makes up the final multi‐tissue aging clock. (b, c) Cross‐validation performance of MultiTIMER given as mean absolute error in years and *R*‐squared. (d) Scatter plot of actual versus predicted age for all training samples. (e) Heatmap of six primary aging phenotypes defined by their activity across all processes in MultiTIMER. Higher/Lower values (red/blue) correspond to an older/younger phenotype. Depicted are the cluster centers that were identified using k‐means clustering.

The selection of cellular processes that are predictive of chronological age resides on the unbiased selection of predictive genes from the set of all genes belonging to any of the 27 processes using a generalized linear model (GLM), which is trained on more than 3000 transcriptomic samples with available chronological age information in the Gene Expression Omnibus (Clough & Barrett, 2016; Figure 1a). Indeed, GLMs constitute an ideal modeling framework, since they can resemble the approximately normal distribution of biological age around chronological age (Belsky et al., 2015). As a result, we identified 818 genes across 25 top‐level processes (Figure S1, Table S1).

### 2.2 Validation of MultiTIMER on chronological age information

Since chronological and biological age are intrinsically related, we first sought to assess the performance of MultiTIMER by validating its ability to predict the chronological age of healthy, non‐disease cells. In this regard, we performed 10‐fold cross validation of the training dataset and showed the high predictive ability of MultiTIMER having a mean absolute error of 8.2 years and an *r*‐squared of 0.75 (Figure 1b–d). In addition, we assessed the activity of individual processes to interrogate whether cell type specific functions govern the age predictions. In this regard, we define process activity as the sum of coefficients weighted by the gene expression for each process. Due to the varying number of genes in each process, our activity measure is scaled to ensure comparability between processes. In this regard, we collected more than 70,000 transcriptional profiles from the Gene Expression Omnibus that are available in the ArchS4 database and annotated them with cell and tissue type information from the Cell Ontology (Diehl et al., 2016) and Tissue Ontology (Gremse et al., 2011) resulting in approximately 23,000 annotated samples. Subsequently, we applied MultiTIMER to these annotated samples and, as a result, observed that functions related to specific cell types are indeed showing a younger phenotype (Figure S2). For instance, neurons show a young phenotype of the “membrane transport of small molecules and electrical properties of membranes” process whereas sperm cells exhibit a young phenotype of “DNA replication, recombination and repair,” “cellular protrusion organization,” and “regulated cell death.” Moreover, we observed two groups of processes that age similarly (Figure S3). The first group is composed of processes related to cell–environment interactions, such as “cellular communication” and “cell junction dynamics.” The second group, in contrast, consists of two brain‐related processes, that is, “neurotransmission” and “membrane transport of small molecules and electrical properties of membranes.” Despite process‐specific enrichment in younger or older processes, we observed in these samples that aging phenotypes can be categorized in six different groups, each corresponding to a unique combination of co‐occurring process activities (Figure 1e). This finding suggests that the aging process is constrained to defined aging phenotypes.

### 2.3 MultiTIMER captures trends of biological aging of cells

**FIGURE 2:**
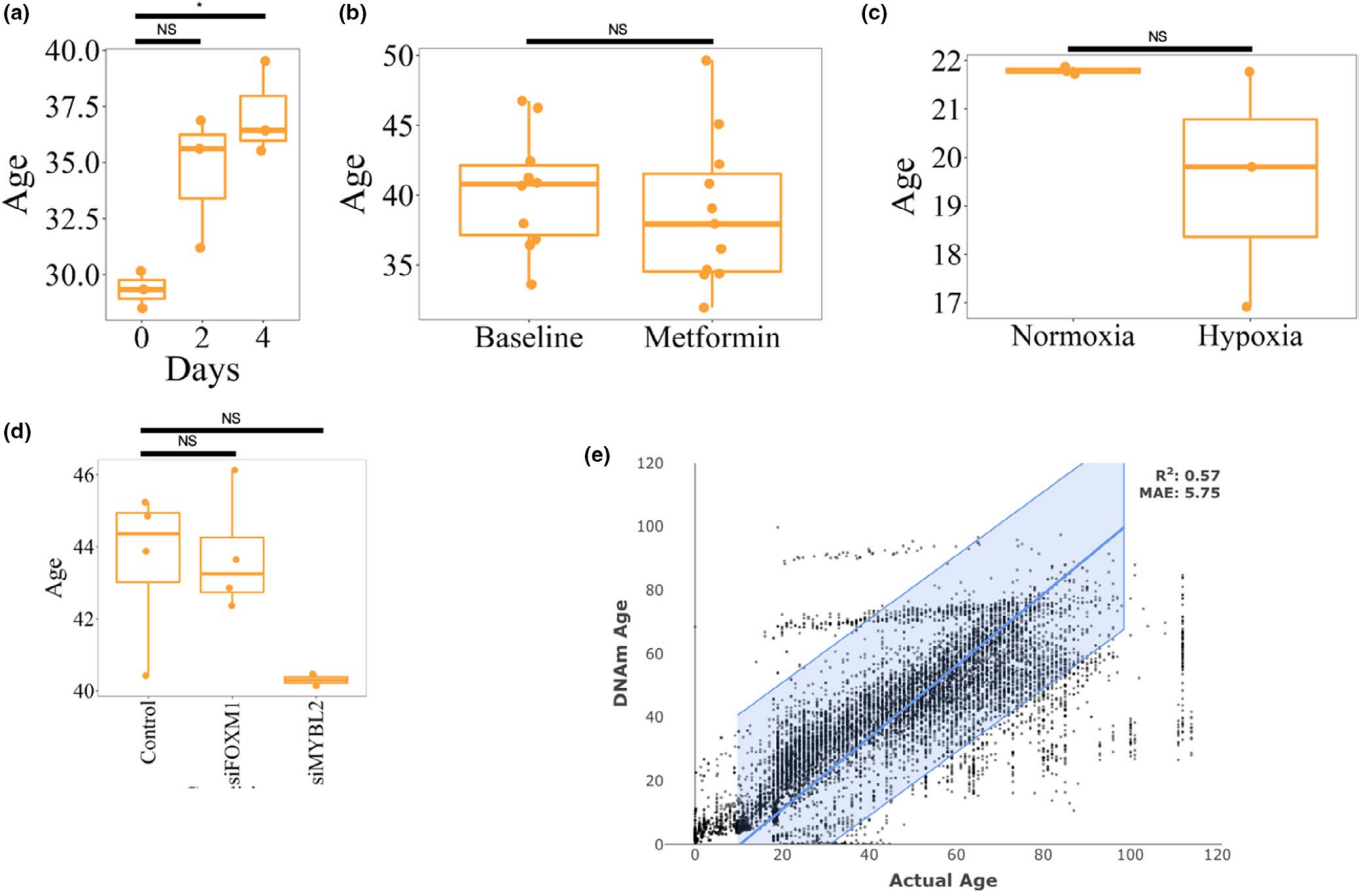
MultiTIMER response to interventions. (a) Treatment with high‐dose rapamycin leads to an increase in apoptosis and a decrease in the proliferation rate, which is reflected in an increased cellular age in fibroblasts (BJ cells) (Yilmaz et al., 2018). Although the predicted age increases already by day two of the treatment [NS], a more homogeneous increase can be observed by day four [*]. (b) Treatment of human healthy volunteers with Metformin shows anti‐inflammatory effects in the context of infections (Lachmandas et al., 2019), but the Interventions Testing Program of the NIA found that it does not promote healthy aging (Partridge et al., 2020). Therefore, we would not expect to see a significant effect on the predicted age in ex vivo blood samples of healthy subjects [NS]. (c) Primary human pericytes cultured in normoxic or hypoxic conditions for 24 h (Bischoff et al., 2017) only show small differences in their predicted age [NS] even though hypoxia is a known cellular rejuvenation treatment. (d) Although FOXM1 inhibition is known to increase the cellular age by inducing a senescent phenotype (Macedo et al., 2018), MultiTIMER does not detect a significant effect in A549 lung adenocarcinoma cells (Macedo et al., 2018). In contrast, MYBL2 has been shown to rejuvenate heart tissue (Rafatian et al., 2020) and is predicted to decrease cellular age in A549 cells although the difference is insignificant [NS] (Mullen et al., 2020). (e) Actual versus DNA‐methylation age in normal, non‐disease samples predicted with the multi‐tissue epigenetic clock (Horvath, 2013). For all statistical comparison, a t‐test has been employed to compute p‐that were adjusted per panel by Benjamini‐Hochberg correction and are reported in brackets (NS: >0.05; *: <0.05; **: <0.005; ***: <0.0005).

Next, we set out to demonstrate that MultiTIMER not only captures chronological age but that the variation we observed in healthy control samples corresponds to different aspects of biological age. For this reason, we collected transcriptomics profiles of cells exposed to 20 cellular stressors, interventions or progeria. These interventions include, for instance, repurposed drugs, such as rapamycin and metformin, as well as stressors, namely hypoxia or induced senescence. As a result, we observed that the predictions in most cases resembles the expected trend of the intervention (Figure 2a–c; Figure S4a,b; Data S2). For instance, rapamycin treatment has been shown to increase the apoptotic rate of fibroblasts and reduces proliferation in the early days of the treatment (Yilmaz et al., 2018), which is reflected in a significantly increased predicted age. Interestingly, Yilmaz et al. demonstrate that the response to rapamycin treatment is highly dependent on the cell type. Moreover, we interrogated which processes in MultiTIMER became younger/older after rapamycin (Figure S8). For instance, rapamycin has been shown to reduce protein translation and thereby improves the protein folding and degradation machinery (Bjedov & Rallis, 2020). Consistently, we observed a rejuvenation in the “intracellular degradation pathways” process that is part of MultiTIMER. In addition, the rejuvenating effects of rapamycin are also reflected in MultiTIMER, showing a younger age for the “cell cycle and cell division” process (Fingar et al., 2004). In contrast, the observed overall increase in cellular age appears to be driven by the “cellular communication,” “posttranslational protein modification,” and “membrane transport of small molecules and electrical properties of membranes” processes. On the other hand, metformin did not alter the predicted age, which is in accordance with previous reports from NIA's Interventions Testing Program (Partridge et al., 2020). In addition, bypassing cellular senescence, one of the hallmarks of aging (López‐Otín et al., 2013), shows a slight yet non‐significant rejuvenation compared to BRAF induced senescence (Figure S4b). Nevertheless, MultiTIMER did not respond to certain interventions, such as Foxm1 inhibition, which supposedly increases the biological age of cells (Macedo et al., 2018; Figure 2d). However, although MultiTIMER is able to correctly resemble the expected trend for most interventions, the observed differences are not significant except for rapamycin (Figure 2a) and quiescence (Figure S4b). Although quiescence has been largely considered to be rejuvenating cells, the observed increased cellular age predicted by MultiTIMER is largely mediated by the “DNA replication, recombination and repair” process (Figure S9). Indeed, error‐prone DNA repair mechanisms in quiescent cells have been shown to have detrimental effects (Mohrin et al., 2010). For instance, overexpression of telomerase reverse transcriptase (hTERT), is expected to increase cellular age. Although the predicted age of the treated cells increases, the observed change is insignificant (Figure S4a). Similarly, treatment of the cells with simian virus 40 (SV) or RAS are supposed to increase cellular age. However, an insignificant reduction of cellular age is predicted instead (Figure S4a). In many cases, the low number of samples hinders the determination of statistical significance although large differences in the predicted age with low variation are observed. For instance, the average predicted age difference between control fibroblasts and hTERT‐transformed fibroblasts is almost 15 years (Figure S4a). Nevertheless, statistical significance cannot be determined due to the presence of only two samples per condition. In addition, some of the interventions may not be mediated by processes that are part of MultiTIMER such that it fails to detect their effects.

### 2.4 Comparison to the epigenetic clock

Due to the prevalence of epigenetic clocks for assessing cellular age, we sought to compare MultiTIMER to the widely used epigenetic multi‐tissue clock proposed by (Horvath, 2013). In this regard, we collected epigenetic profiles of both healthy cells as well as interventions with known effects on cellular functions and applied the multi‐tissue clock. In contrast to MultiTIMER, the epigenetic clock shows a lower accuracy in predicting chronological age (Figures 1b and 2e; MultiTIMER: *R* ^2^ = 0.75, Epi: *R* ^2^ = 0.54). Interestingly, considering the overall test set performance of *R* ^2^ = 0.92 of the epigenetic clock after training, its performance appears to be more variable then initially assumed. Moreoever, we observed differences in capturing biological age by MultiTIMER and the epigenetic clock. In particular, the epigenetic clock captures the correct trend of the expected effect in only 10 out of 21 (48%) interventions (Figure S5). In addition, although the average number of available samples per condition is larger compared to RNA‐seq samples, statistical significance of the predicted age differences can only be established for replication inhibition of fibroblasts compared to control cells. The observed differences in the response can be attributed to MultiTIMER's more direct relation of functional processes with the predicted age of the cells whereas the multi‐tissue epigenetic clock considers methylated CpG that are scattered across the genome and whose effect largely remains elusive.

### 2.5 Comparison to another transcriptional age predictor

We finally sought to compare MultiTIMER to RNAAgeCalc (Ren & Kuan, 2020), a multi‐tissue age predictor that is based on RNA‐seq data. We applied RNAAgeCalc to the normal, non‐disease samples used for training MultiTIMER as well as to the interventions we previously assessed (Figure S6). With respect to normal samples, RNAAgeCalc with its default gene signature shows low performance with a mean absolute error (MAE) of almost 35 years and an *r*‐squared of 0.18 (Figure S6a). Although the performance varies when using different gene signatures for the prediction, the predictive power in these samples remains low (*r*‐squared range: 0–0.43; MAE range: 22.82–34.83) (Figure S7; Data S3). Similar to MultiTIMER, the predicted age differences when comparing control and treatment samples is insignificant for almost all tested interventions. In contrast to MultiTIMER, RNAAgeCalc only shows a significant difference of the cellular age that agrees with the expected effect in response to hypoxia (Figure S6d). Moreover, the number of significant differences remains low even when considering different gene signatures for the age prediction (range: 1–5) (Data S3). In case of rapamycin and MYBL2 inhibition, we detected a significant effect that is contrary to the expected effect of the intervention (Figure S6b,g). In addition, although insignificant, the predicted effect upon Foxm1 inhibition is not resembling the known effect of the intervention. Interestingly, when assessing hypoxia in a dataset of fibroblast samples, RNAAgeCalc predicts an increase of cellular age in response to the treatment (Figure S6e). Overall, RNAAgeCalc detects a significant effect in response to hypoxia in pericytes that remains undetected by MultiTIMER. However, the response to multiple interventions is contrary to their expected effects. Thus, we believe MultiTIMER to be of great utility in characterizing the aging process and will enable large‐scale screens for novel rejuvenating interventions.

## 3 MATERIALS AND METHODS

### 3.1 Collection and selection of RNA‐seq samples

Gene counts of human RNA‐seq samples have been obtained from ArchS4 v11 (Lachmann et al., 2018) and further filtered based on the following criteria: (i) the probability of the sample to be from a single cell is less than or equal to 0.1, (ii) the number of aligned reads is greater than 10 million, (iii) the library selection method is “cDNA,” (iv) the library strategy is “RNA‐seq,” (v) the library source is “transcriptomic,” (vi) the organism is “Homo sapiens,” (vii) the molecule is “total RNA,” and (viii) the taxonomy id is “9606.”

Each sample was annotated based on its Gene Expression Omnibus (Clough & Barrett, 2016) metadata and further filtered based on its “source” and “characteristic” fields to those include selected annotations from the Cell and Tissue Ontologies (Diehl et al., 2016; Gremse et al., 2011). In particular, samples that include terms related to other species have been excluded. Moreover, training samples have been selected based on the following criteria: (i) their “source” or “characteristic” metadata contains one of the terms “control,” “healthy,” “WT,” “wildtype,” “untreated,” “normal,” or “Treatment: None” (case insensitive) and (ii) their “characteristic” or “source” metadata contains a numeric age in years, that is, that cannot be related to months, days, or embryonic development.

### 3.2 RNA‐seq data preprocessing

Gene counts of selected samples were normalized using edgeR's TMM with singleton pairing normalization (Robinson et al., 2010). After normalization, we removed batch effects using ComBat from the sva R package (Leek et al., 2012). Samples having the same GSE Id were considered to come from the same batch. Finally, genes having a mean corrected expression of less than −3.8 across all samples were removed.

### 3.3 DNA‐methylation data collection and DNAm age prediction

DNA‐methylation data fulfilling the following conditions has been obtained from Gene Expression Omnibus (Clough & Barrett, 2016): (i) It has been generated using HumanMethylation25, HumanMethylation450 or HumanMethylation850 arrays, (ii) it contains information about the chronological age of the samples and (iii) the sample is from a healthy, non‐disease cell source. DNA‐methylation samples of interventions have been manually mined by searching for the name of the intervention in Gene Expression Omnibus (Clough & Barrett, 2016).

DNAm age predictions have been performed using “DNAmAge” function in the methylclock R package (Pelegí‐Sisó et al., 2021). In particular, DNAm age was predicted based on Horvath's multi‐tissue clock (Horvath, 2013) and, in case the data was provided as M values, measurements were transformed to beta values. Only samples with more than 50% overlap of measured CpGs with clock CpGs were considered.

### 3.4 Training MultiTIMER

Training of our functionally interpretable multi‐tissue RNA clock, MultiTIMER, requires a three‐step approach. First, gene‐function associations were obtained from the MBotC (Hansen et al., 2017). Second, transcriptomic control samples with associated chronological age information are obtained (as described before) and subset to all genes in the 27 top‐level processes in the MBotC (process level 1). Lastly, all genes associated with these processes are supplied to a GLM with 10‐fold cross‐validation, gaussian distribution, identity link function, and elastic net regularization (parameter alpha = 0.5). Model fitting is performed using the R package h2o (H2O.ai, n.d.) and the distribution family to use is automatically determined by the fitting approach.

### 3.5 Calculating process activity and definition of aging phenotypes

After training MultiTIMER, we define the activity of each selected process in each sample. Process activity is related to the age of a sample when only the genes of a single process are used for predicting the age. In particular, we define process activity as the sum of the model coefficients weighted by their corresponding gene expression levels. Thus, in contrast to the predictions of MultiTIMER, process activity does not consider the transformation of distribution family and its associated link function. Nevertheless, higher process activity values correspond to a higher age (higher dysregulation) whereas lower values reflect less dysregulation and therefore a lower age. Thus, this measure serves as a surrogate for assessing the level of dysregulation of each process in each sample.

When applied to the subset of 23,000 transcriptomic samples with cell or tissue type annotation, we clustered samples based on their process activity. To determine the optimal number of clusters, we computed the gap statistic for k‐means clustering using the clusGap function of the cluster package in R (Maechler et al., 2022). In this regard, we assessed clusterings in the range of one to 24 using 100 bootstrap samples (parameter B) and 50 restarts of the k‐means algorithm per number of clusters (parameter nstart).

### 3.6 Comparison of MultiTIMER with RNAAgeCalc

MultiTIMER predictions were compared with RNAAgeCalc predictions using the ‘predict_age’ function of the RNAAgeCalc R package v1.10.0. Expression data was provided as raw counts (indicated by setting the exprtype parameter to “counts”) with ID‐type “SYMBOL.” To achieve a fair comparison, the cross‐tissue predictor of RNAAgeCalc that is agnostic of race has been selected by leaving the tissue parameter empty and setting the stype parameter to “all.” The signature parameter has been varied to observe variability in the predictions. When using the cross‐tissue predictor, the “DESeq2” signature is not available and has therefore been excluded. All other parameters were invoked with default values.

## AUTHOR CONTRIBUTIONS

S.J. implemented MultiTIMER, performed analyses, created figures, and wrote the manuscript. J.A.H. performed analyses, A.d.S. conceived the idea, discussed the analyses and results, and wrote the manuscript.

## CONFLICT OF INTEREST STATEMENT

None declared.

## Supporting information


Appendix S1.

Data S1.

Data S2.

Data S3.

Table S1.

## Data Availability

All data are available in the main text or the supplementary materials. Data S1 contains lists of all datasets used in this study. The code for MultiTIMER can be found in Github (https://github.com/saschajung/MultiTIMER).
