# Supplementary material for: Measuring biological age using a functionally interpretable multi‐tissue RNA clock
Source: Aging Cell. 2023 Mar 16;22(5):e13799. doi: 10.1111/acel.13799 (PMC10186600; doi:10.1111/acel.13799)
Supplement: Supplementary file 1 — Appendix S1. [file ACEL-22-e13799-s003.docx]

**
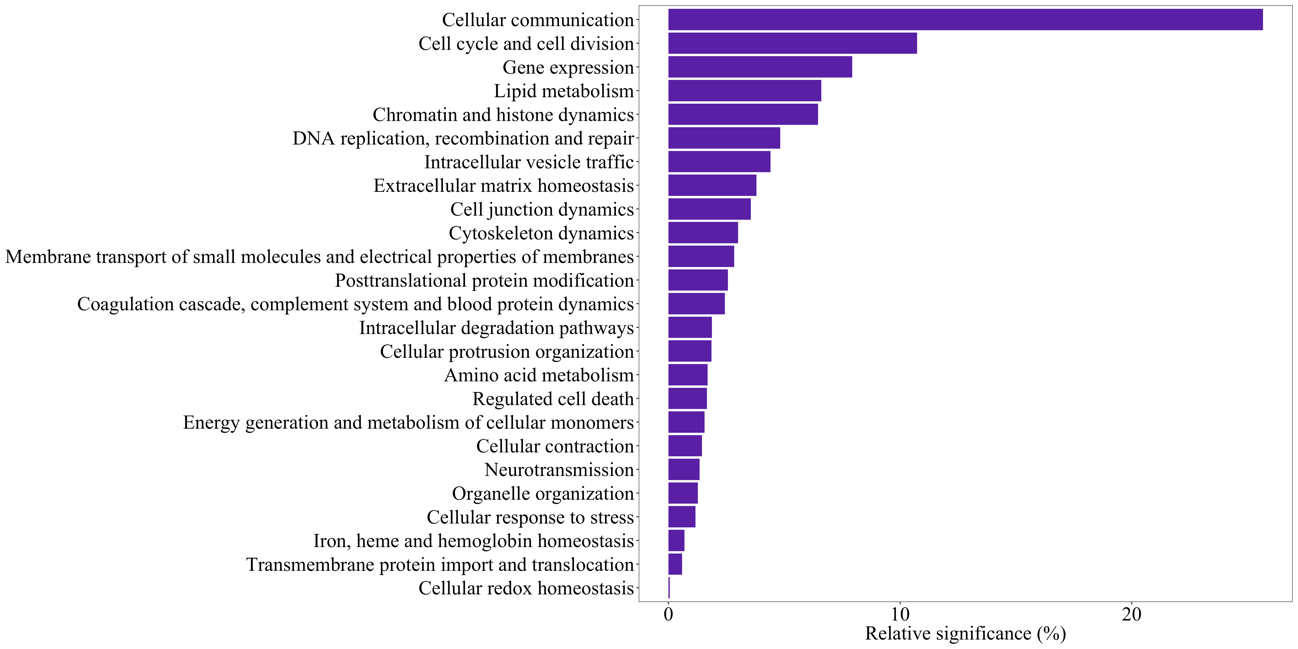
**

**Figure S1. Relative significance of MultiTIMER processes.** Significance is computed by summing the absolute coefficients of all genes belonging to each process. Significance values are normalized to sum to 100%.


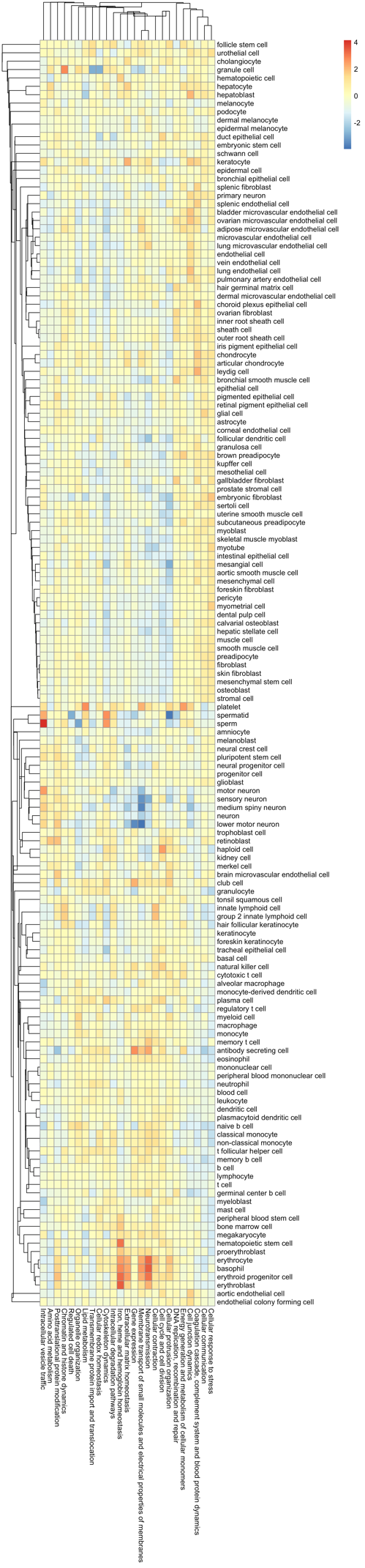


**Figure S2. Process activity of different cell types.** Heatmap of cell type process activity across all 25 processes in MultiTIMER (Activities of samples of the same cell type have been averaged). Here, activity is defined as the sum of coefficients weighted by the gene expression for each process. Higher values (red) correspond to an older phenotype whereas lower values (blue) correspond to a younger phenotype.


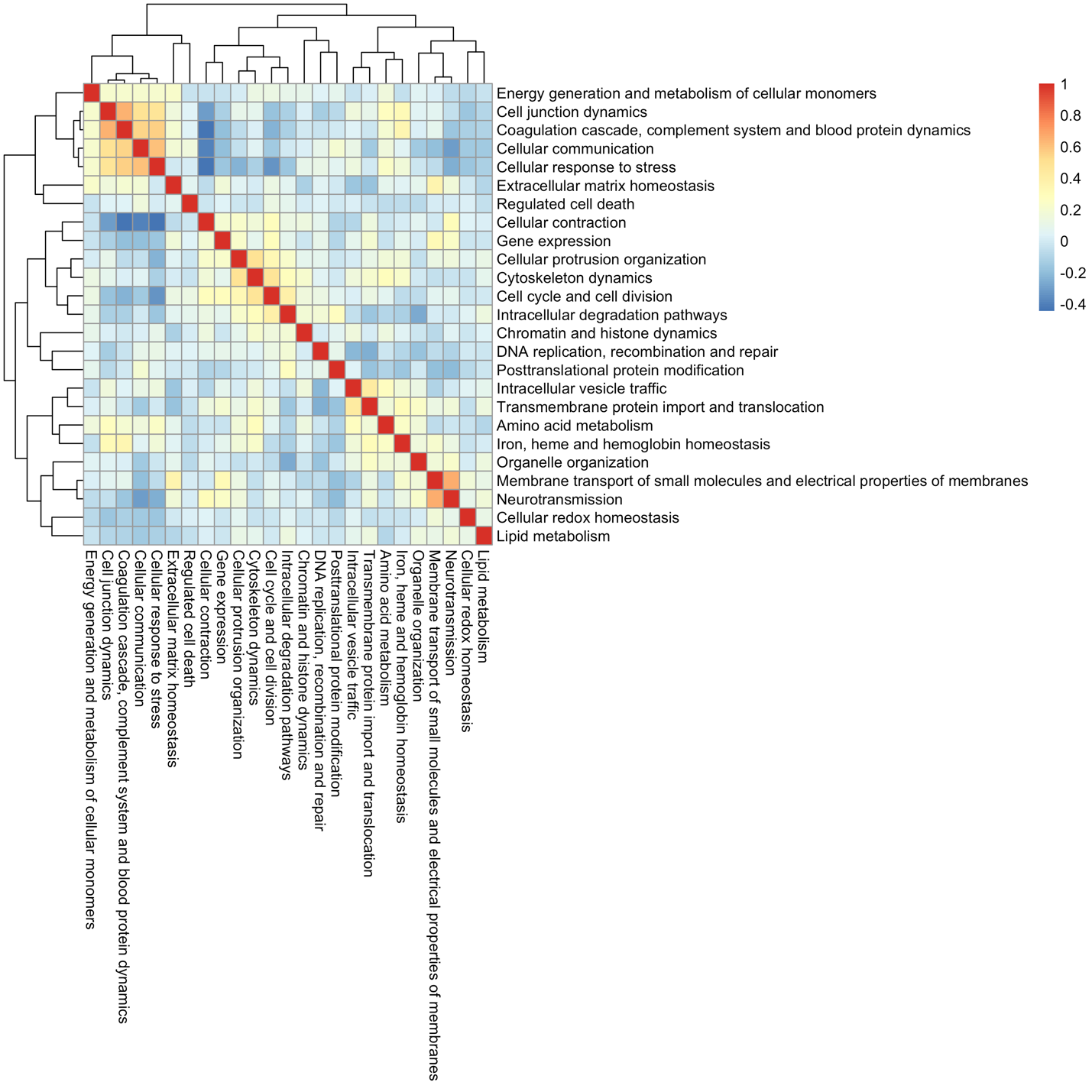


**Figure S3. Correlation of process activity.** Pearson correlation of process activities across 70000 RNA-seq samples has been calculated. As a result, we observe two highly correlated clusters of processes that age similarly. The first cluster is comprised of cell-environment interaction processes including “Cell junction dynamics”, “Cellular Communication”, “Coagulation cascade” and “Cellular response to stress”. In contrast, the second cluster is comprised of “Neurotransmission” and “Membrane transport and electrical properties of membranes”.

**Figure S4. MultiTIMER response to additional interventions.** MultiTIMER age predictions for 6 interventions and corresponding controls in (**a**) BJ and (**b**) Tig3 diploid lung fibroblasts (refs. (Danielsson et al. 2018; Lenain et al. 2017)). Control samples are termed “None”, “Cycling” or “BRAF Senescence Bypass). Interventions can be classified into two categories as described below. A t-test was performed to assess the significance of the predicted effect when comparing interventions to control samples. P-values have been adjusted per panel by Benjamini-Hochberg correction and are reported in brackets (NS: >0.05; *: < 0.05; **: < 0.005; ***: < 0.0005). Rejuvenating Interventions: Hypoxia [NS], BRAF Senescence Bypass [NS] and Quiescent [*]. Pro-aging interventions: hTERT [NS], RAS [NS] and SV [NS].

**Figure S5. Response of DNA methylation clock to biological interventions.** DNA methylation age predicted by the epigenetic clock for 21 interventions and corresponding controls in fibroblasts (ref. (Kabacik et al. 2022)) (**A**,**E**), Keratinocytes (ref. (Kabacik et al. 2022)) (**B**), HUVEC cells (ref. (Kabacik et al. 2022)) (**C**) and blood (ref. (Bejaoui et al. 2022)) (**D**). Control samples are termed “Control”, “None” or “Healthy”. Interventions can be classified into two categories, as follows. A t-test was performed to assess the significance of the predicted effect when comparing interventions to control samples. P-values have been adjusted per panel by Benjamini-Hochberg correction and are reported in brackets (NS: >0.05; *: < 0.05; **: < 0.005; ***: < 0.0005). Rejuvenating Interventions: Bezafibrate [NS], Metformin [NS], nicotinamide adenine dinucleotide (NAD) [NS], nicotinamide riboside (NR) [NS], Rapamycin [NS], ASO (Line 1) [NS]. Pro-aging interventions: Chronic Radiation [NS], hTERT [NS], RAS [NS], Replication [***], X-ray [NS], carbonyl cyanide m-chlorophenylhydrazone (CCCP) [NS], Hutchinson-Gilford Progeria Syndrome (HGPS) [NS], Progeroid laminopathies [NS], Werner syndrome [NS].


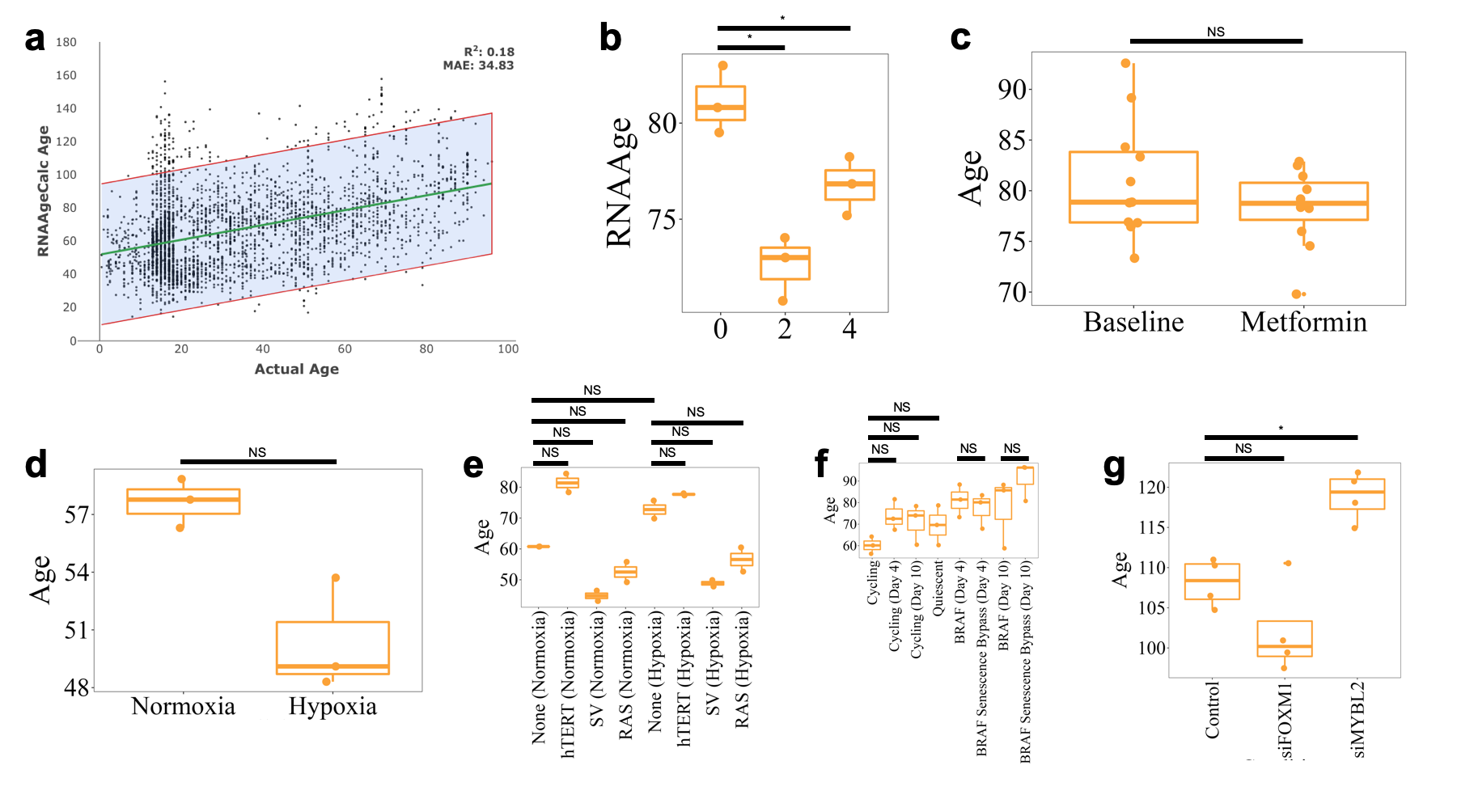


**Figure S6. RNAAgeCalc response to normal samples and interventions.** (a) Actual versus predicted age by RNAAgeCalc in normal, non-disease samples predicted with the multi-tissue predictor. (b) Treatment with high-dose rapamycin leads to an increase in apoptosis and a decrease in the proliferation rate, which is reflected in an increased cellular age in fibroblasts (BJ cells) (ref. (Yilmaz et al. 2018)). However, RNAAgeCalc predicted a significant decrease already by day 2 of the treatment [*] and remains significant at day 4 [*]. (c) Treatment of human healthy volunteers with Metformin shows anti-inflammatory effects in the context of infections(Lachmandas et al. 2019), but the Interventions Testing Program of the NIA found that it does not promote healthy aging (Partridge et al. 2020). RNAAgeCalc does not respond to metformin treatment in ex vivo blood samples of healthy subjects [NS]. (d) Primary human pericytes cultured in normoxic or hypoxic conditions for 24 hours(Bischoff et al. 2017) show a significant reduction in the predicted age [*] in accordance with its expected effect. (e,f) RNAAgeCalc age predictions for 6 interventions and corresponding controls in (e) BJ and (f) Tig3 diploid lung fibroblasts (refs. (Danielsson et al. 2018; Lenain et al. 2017)). Control samples are termed “None”, “Cycling” or “BRAF Senescence Bypass). Interventions can be classified into two categories as follows. Rejuvenating Interventions: Hypoxia [NS], BRAF Senescence Bypass [NS] and Quiescent [NS]. Pro-aging interventions: hTERT [NS], RAS [NS] and SV [NS]. (g) Although FOXM1 inhibition is known to increase the cellular age by inducing a senescent phenotype(Macedo et al. 2018), RNAAgeCalc instead detects a non-significant reduction of age in A549 lung adenocarcinoma cells [NS] (ref. (Macedo et al. 2018)). In contrast, MYBL2 has been shown to rejuvenate heart tissue(Rafatian et al. 2020) but is predicted to increase cellular age significantly in A549 cells [*] (ref. (Mullen et al. 2020)). A t-test was performed to assess the significance of the predicted effects when comparing interventions to control samples. P-values have been adjusted per panel by Benjamini-Hochberg correction and are reported in brackets (NS: >0.05; *: < 0.05; **: < 0.005; ***: < 0.0005).


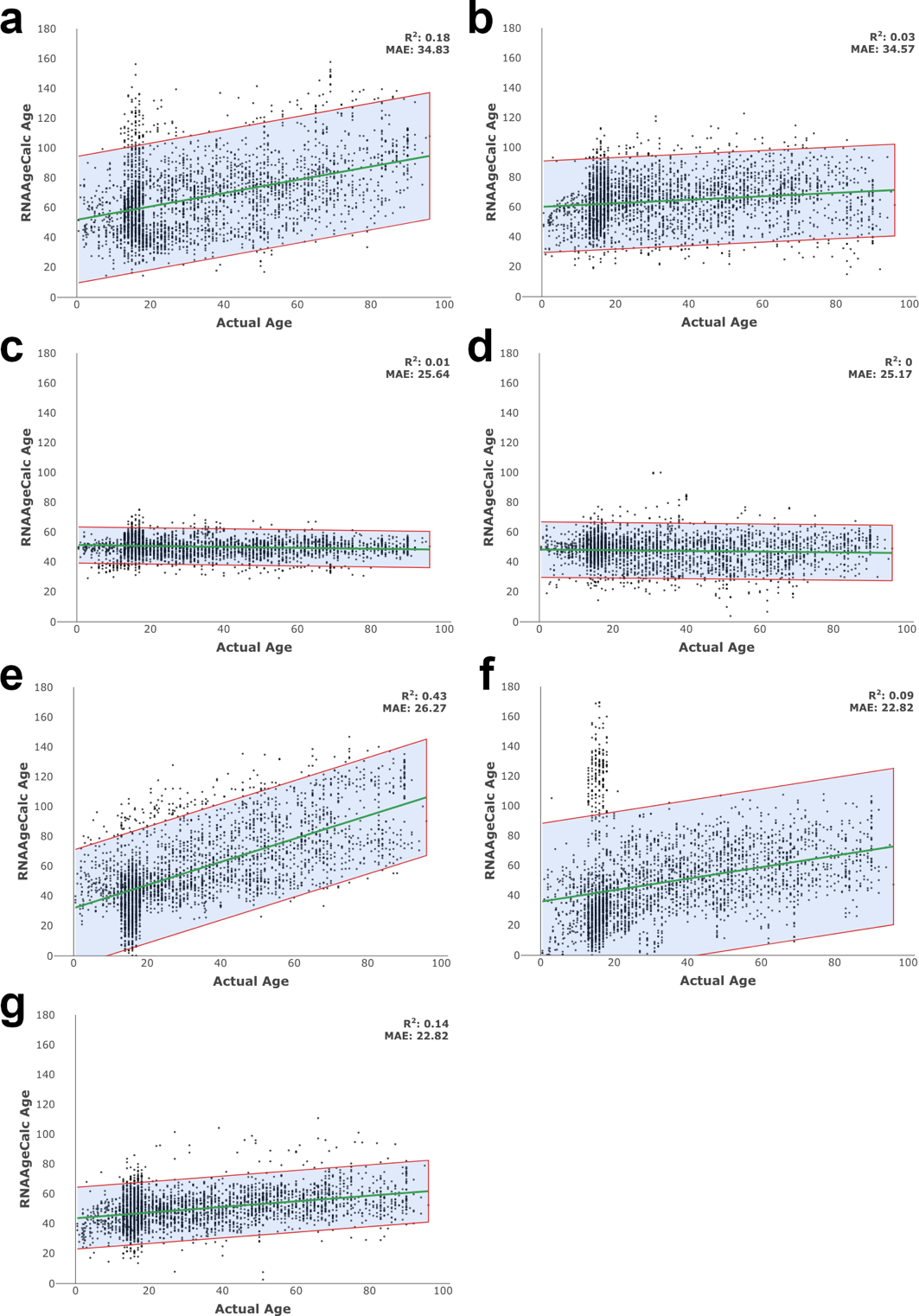


**Figure S7. RNAAgeCalc response to normal samples with various gene signatures.** Actual versus predicted age by RNAAgeCalc using different signature genes for the prediction: (a) Pearson correlation, (b) deviance, (c) deMagalhaes, (d) GenAge, (e) GTExAge, (f) Peters and (g) all signature genes combined. R-squared and mean absolute error (MAE) are indicated in each panel.

**
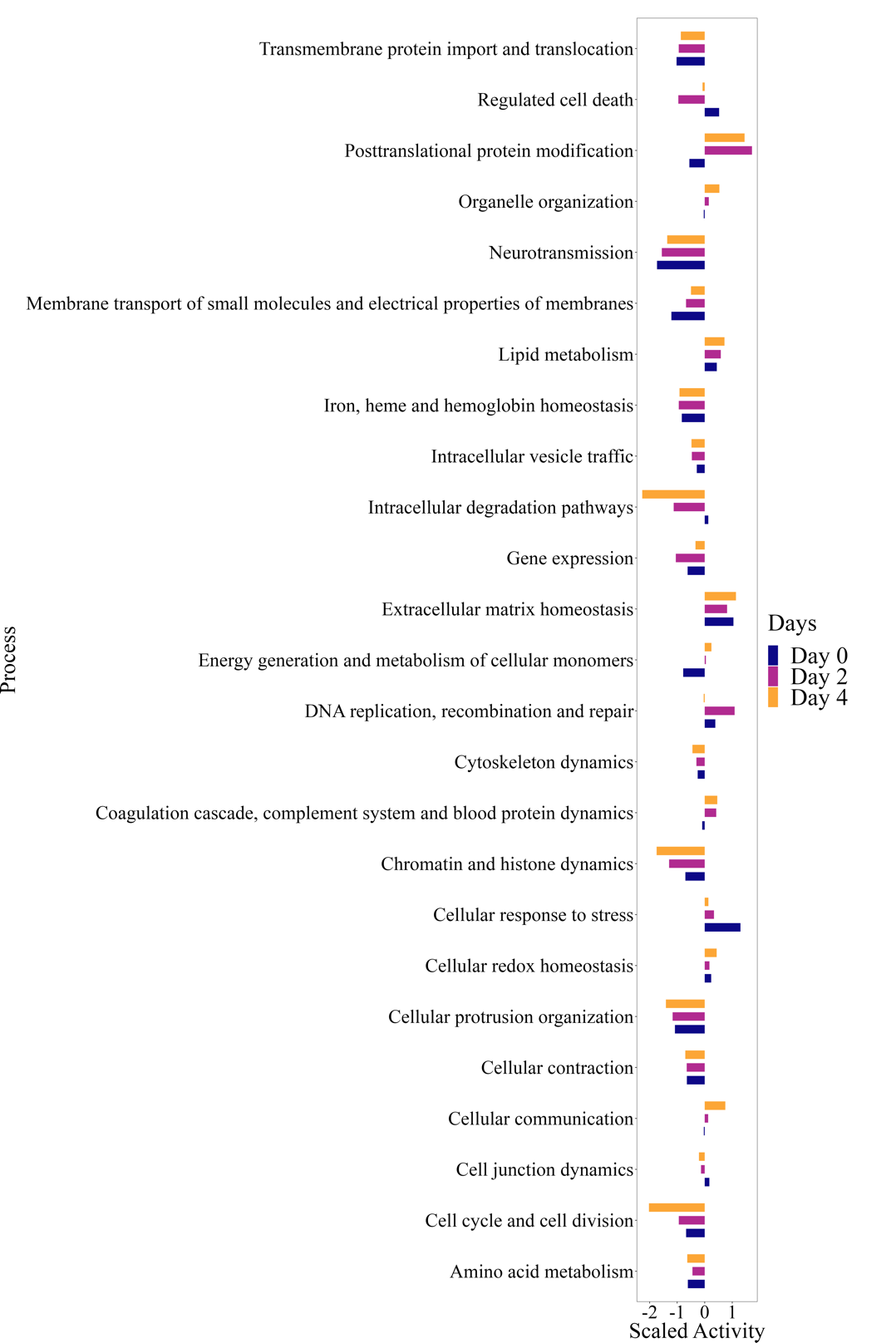
**

**Figure S8. Individual changes in process activity after rapamycin treatment.** Rapamycin treatment of BJ fibroblasts has different effects on individual processes in MultiTIMER. Lower/higher scaled activity values correspond to lower/higher age. Processes such as “Cell cycle and cell division”, “Cellular response to stress”, “Chromatin and histone dynamics” and “Intracellular degradation pathways” become rejuvenated whereas “Cellular communication”, “Membrane transport of small molecules and electrical properties of membranes” and “Posttranslational protein modification” become older. Each bar corresponds to the average process activity across three replicates.

**
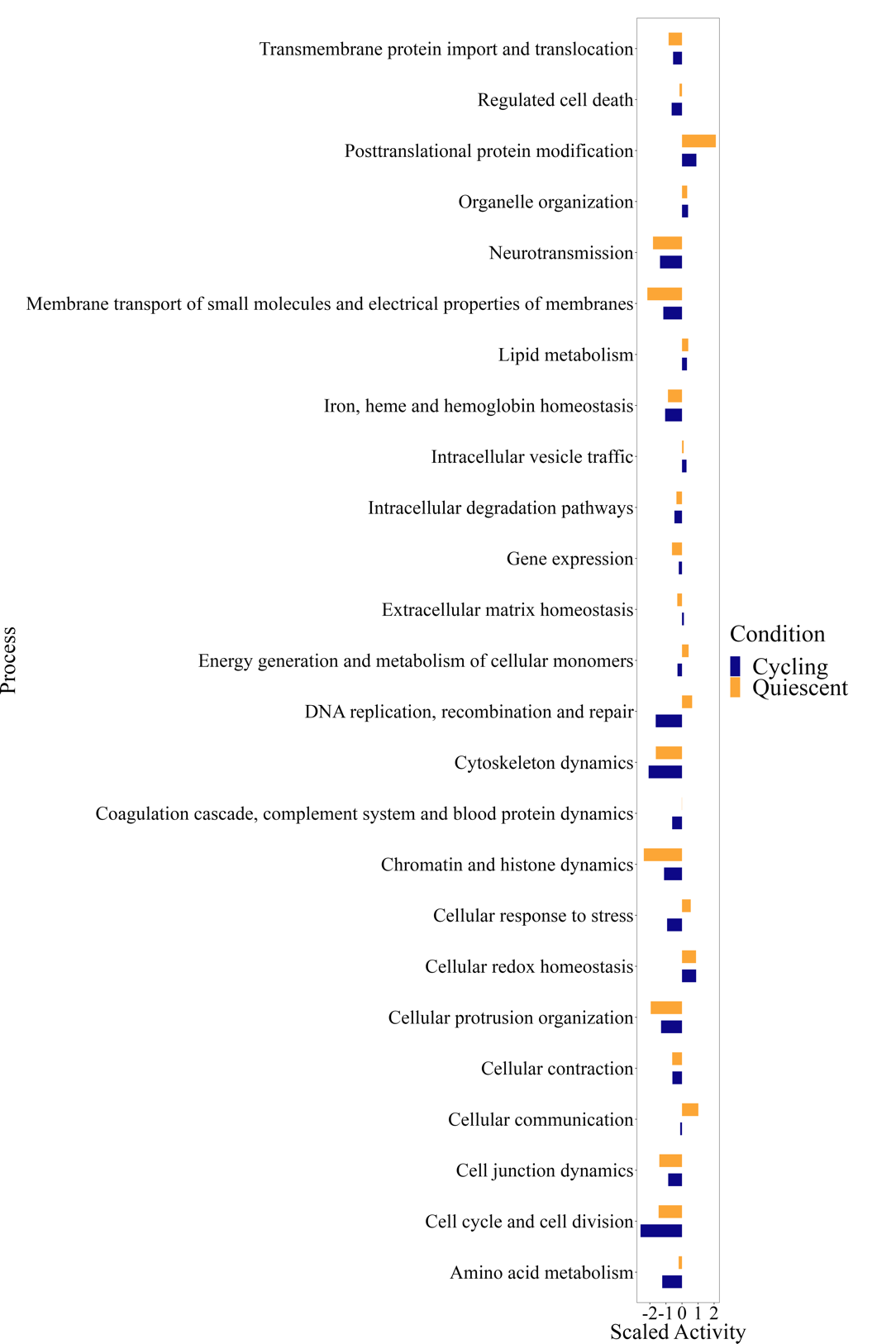
**

**Figure S9. Individual changes in process activity in quiescent cells.** Quiescence of fibroblasts has different effects on individual processes in MultiTIMER. Lower/higher scaled activity values correspond to lower/higher age. The most pronounced difference in process activity can be observed in the “DNA replication, recombination and repair” process, which acquires an older phenotype. Each bar corresponds to the average process activity across three replicates.

**Table S1. Genes Included in MultiTIMER.** The genes (as gene symbols) along with their (non-)standardized weights in MultiTIMER and the processes they belong to.

**Dataset S1. Datasets used in this study.** This dataset contains all datasets that have been used in this study organized by figure numbers.

**Dataset S2. MultiTIMER intervention statistics.** This dataset contains summary statistics of all MultiTIMER predictions in intervention datasets.

**Dataset S3. RNAAgeCalc predictions.** This dataset contains RNAAgeCalc predictions and summary statistics based on different gene signatures.

**Supplementary references**

Bejaoui Y, Razzaq A, Yousri NA, Oshima J, Megarbane A, Qannan A, Potabattula R, Alam T, Martin GM, Horn HF, Haaf T, Horvath S & El Hajj N (2022) DNA methylation signatures in Blood DNA of Hutchinson-Gilford Progeria syndrome. *Aging Cell* 21, e13555. Available at: http://www.ncbi.nlm.nih.gov/pubmed/35045206.

Bischoff FC, Werner A, John D, Boeckel J-N, Melissari M-T, Grote P, Glaser SF, Demolli S, Uchida S, Michalik KM, Meder B, Katus HA, Haas J, Chen W, Pullamsetti SS, Seeger W, Zeiher AM, Dimmeler S & Zehendner CM (2017) Identification and Functional Characterization of Hypoxia-Induced Endoplasmic Reticulum Stress Regulating lncRNA (HypERlnc) in Pericytes. *Circ. Res.* 121, 368–375. Available at: http://www.ncbi.nlm.nih.gov/pubmed/28611075.

Danielsson F, Fasterius E, Sullivan D, Hases L, Sanli K, Zhang C, Mardinoglu A, Al-Khalili C, Huss M, Uhlén M, Williams C & Lundberg E (2018) Transcriptome profiling of the interconnection of pathways involved in malignant transformation and response to hypoxia. *Oncotarget* 9, 19730–19744. Available at: http://www.ncbi.nlm.nih.gov/pubmed/29731978.

Kabacik S, Lowe D, Fransen L, Leonard M, Ang S-L, Whiteman C, Corsi S, Cohen H, Felton S, Bali R, Horvath S & Raj K (2022) The relationship between epigenetic age and the hallmarks of aging in human cells. *Nat. Aging* 2, 484–493. Available at: https://www.nature.com/articles/s43587-022-00220-0.

Lachmandas E, Eckold C, Böhme J, Koeken VACM, Marzuki MB, Blok B, Arts RJW, Chen J, Teng KWW, Ratter J, Smolders EJ, Van den Heuvel C, Stienstra R, Dockrell HM, Newell E, Netea MG, Singhal A, Cliff JM & Van Crevel R (2019) Metformin Alters Human Host Responses to Mycobacterium tuberculosis in Healthy Subjects. *J. Infect. Dis.* 220, 139–150. Available at: http://www.ncbi.nlm.nih.gov/pubmed/30753544.

Lenain C, de Graaf CA, Pagie L, Visser NL, de Haas M, de Vries SS, Peric-Hupkes D, van Steensel B & Peeper DS (2017) Massive reshaping of genome-nuclear lamina interactions during oncogene-induced senescence. *Genome Res.* 27, 1634–1644. Available at: http://www.ncbi.nlm.nih.gov/pubmed/28916540.

Macedo JC, Vaz S, Bakker B, Ribeiro R, Bakker PL, Escandell JM, Ferreira MG, Medema R, Foijer F & Logarinho E (2018) FoxM1 repression during human aging leads to mitotic decline and aneuploidy-driven full senescence. *Nat. Commun.* 9, 2834. Available at: http://www.ncbi.nlm.nih.gov/pubmed/30026603.

Mullen DJ, Yan C, Kang DS, Zhou B, Borok Z, Marconett CN, Farnham PJ, Offringa IA & Rhie SK (2020) TENET 2.0: Identification of key transcriptional regulators and enhancers in lung adenocarcinoma. *PLoS Genet.* 16, e1009023. Available at: http://www.ncbi.nlm.nih.gov/pubmed/32925947.

Partridge L, Fuentealba M & Kennedy BK (2020) The quest to slow ageing through drug discovery. *Nat. Rev. Drug Discov.* 19, 513–532. Available at: http://www.ncbi.nlm.nih.gov/pubmed/32467649.

Rafatian G, Kamkar M, Parent S, Michie C, Risha Y, Molgat ASD, Seymour R, Suuronen EJ & Davis DR (2020) Mybl2 rejuvenates heart explant-derived cells from aged donors after myocardial infarction. *Aging Cell* 19, e13174. Available at: http://www.ncbi.nlm.nih.gov/pubmed/32558221.

Yilmaz A, Peretz M, Aharony A, Sagi I & Benvenisty N (2018) Defining essential genes for human pluripotent stem cells by CRISPR-Cas9 screening in haploid cells. *Nat. Cell Biol.* 20, 610–619. Available at: http://www.ncbi.nlm.nih.gov/pubmed/29662178.
